# Effects of Acute Exposure to Polystyrene Nanoplastics on the Channel Catfish Larvae: Insights From Energy Metabolism and Transcriptomic Analysis

**DOI:** 10.3389/fphys.2022.923278

**Published:** 2022-06-01

**Authors:** Qichen Jiang, Xiaohui Chen, Hucheng Jiang, Minghua Wang, Tongqing Zhang, Wenyi Zhang

**Affiliations:** ^1^ Freshwater Fisheries Research Institute of Jiangsu Province, Nanjing, China; ^2^ Institute of Animal Genetic Resource, Nanjing Normal University, Nanjing, China

**Keywords:** energy metabolism, fish larvae, nanoplastics, proteasomes and HSPs, transcriptomic response

## Abstract

Microplastics (nanoplastics) pollution has been a major ecological issue threatening global aquatic ecosystems. However, knowledge of the adverse effects of nanoplastics and the effects on freshwater ecosystems is still limited. To understand the impacts of nanoplastics on freshwater ecosystems, it is essential to reveal the physiological changes caused by nanoplastics in freshwater organisms, especially at their early life-history stages. In the present study, the larval channel catfish *Ietalurus punetaus* were exposed to gradient concentrations (0, 5, 10, 25, and 50 mg/L) of 75-nm polystyrene nanoplastics (PS-NPs) for 24 h or 48 h, and changes in contents of energy metabolites, metabolic enzyme activities and transcriptome were assessed. The results showed that glucose and triglyceride contents increased after 24 h of exposure to 10 or 25 mg/L of PS-NPs but decreased with increased concentrations or prolonged exposure duration. Activities of most metabolic enzymes analyzed decreased in the larvae after 48 h of exposure, especially in 25 or 50 mg/L of PS-NPs. These suggested that PS-NPs caused huge energy consumption and disturbed the energy metabolism in larval fish. Transcriptomic analysis showed that 48 h of exposure to 50 mg/L PS-NPs affected the expression of genes involved in protein digestion and induced response of proteasomes or heat shock proteins in the larval *I. punetaus*. The genes involved in peroxisome proliferator-activated receptors (PPAR) pathway and biosynthesis of amino acids were activated after the exposure. PS-NPs also depressed the expression of the genes involved in gonad development or muscle contraction in the larval *I. punetaus*. Overall, acute exposure to 75-nm PS-NPs disrupted the energy metabolism by consuming the energy reserves, and affected a series of molecular pathways which may further affect the development and survival of fish. This study provided the information about adverse effects of nanoplastics on the fish larvae and revealed the molecular pathways for the potential adverse outcomes.

## Introduction

Plastic pollution has been a serious problem threatening global environmental security with increased usage and production of plastic products (PlasticsEurope, 2018). Due to the high persistence, plastic waste can accumulate in different environments, from aquatic to soil environments (Zhu et al., 2018; Barría et al., 2020). Plenty of plastics debris ends up in waterways and is transported into the ocean (Lonnstedt and Eklov, 2016). Plastics can be broken into smaller fragments, including microplastics (MPs, diameter 0.1 µm–5 mm) or nanoplastics (NPs, diameter <0.1 µm), and these two forms can be ingested by organisms and be transferred along food chains (Cole et al., 2013; Cole and Galloway, 2015).

Ingestion of microplastics can cause blockage of the digestive tract and lead to reduced energy reserves, metabolic depression, or behavioral alteration of organisms (Wright et al., 2013; Yin et al., 2018; Yin et al., 2019). Compared with MPs, NPs can cause more damage to organisms (González-Fernández et al., 2018). NPs can be more easily ingested by organisms because of their physical properties including smaller size and larger surface-to-volume ratio and can remain in the bodies of organisms for a longer time (Ward and Kach, 2009; Kühn et al., 2015). Once entering organisms, NPs penetrate cells and encounter more complex biological fluids (Mattsson et al., 2015; Canesi et al., 2017). Afterward, NPs further interact with proteins and impair multiple biological functions of organisms, such as lipid metabolism, immune system, reproduction, or blood coagulation (Martin et al., 2008; Zhang et al., 2011; Zhang et al., 2019). However, knowledge of the adverse effects of NPs is still not completely clear and studies on physiological processes, stress responses and molecular mechanisms in organisms during exposure to NPs are needed to further understand the toxicity of NPs.

In past decades, studies about the impacts of MPs or NPs on marine organisms have received much attention while knowledge of the adverse effects of these plastics on freshwater organisms and freshwater ecosystems is still in infancy (Li C. et al., 2020). Plastic pollution is released in the urban area and reaches rivers and, therefore, the freshwater environments provide the sources of marine plastics pollution (Rech et al., 2014). One field study has reported that several freshwater fish ingested small plastics from the river (Sanchez et al., 2014). In aquatic ecosystems, fish are at the higher trophic levels of food chains and more small plastic particles can be accumulated in fish. Fish can ingest MPs or NPs directly or *via* preying on other organisms which accumulate MPs or NPs (Mattsson et al., 2015; Horton et al., 2018). In addition, one study on zebrafish *Danio rerio* showed that small plastics can enter the offspring through the parental gametes (Pitt et al., 2018). Fish larvae are more vulnerable to environmental pollutants due to their small size and immaturity compared with adults or juveniles, and their development and survival determine the physiological traits of the adult stage and the population health. Therefore, knowledge of the effects of NPs on the physiological processes of fish larvae is essential for further understanding the consequences of plastic pollution.

The present study aimed to reveal the effects of acute PS-NPs exposure on larval fish. The larvae of the channel catfish *Ietalurus punetaus*, which is a large omnivorous fish and an important aquaculture species, were exposed to gradient concentrations of polystyrene nanoplastics (PS-NPs) for 24 or 48 h. Polystyrene plastic, which is a ubiquitously distributed plastic in aquatic environments and makes up about 6.7% of total plastic products, was selected to perform NPs exposure (Hidalgoruz et al., 2012). After the exposure, the effects of NPs exposure on energy metabolites, metabolic enzyme activities and antioxidant defense of *I. punetaus* larvae were assessed. Changes in the transcriptome of the larval fish were analyzed after 48 h of exposure to PS-NPs to reveal the potential molecular pathways of the effects of PS-NPs.

## Materials and Methods

### Organisms and Polystyrene Nanoplastics Exposure

The unlabeled 75-nm PS-NPs particles used in the present study were purchased from Baseline Chromtech Research Centre, Tianjin, China. The primary diameter, size distribution and ze-ta-potential of polystyrene nanoplastics were determined as our previous studies (Liu et al., 2018a; Liu et al., 2018b). The PS-NPs were stable in water and no aggregation was observed (Supplementary Material S1).

The channel catfish *Ietalurus punetaus* larvae (mass = 0.222 ± 0.015 g) were obtained from the National Genetics and Breeding Center of Channel Catfish (Nanjing, P.R. China) and were reared in tanks with aerated tap water at 26°C, a dissolved oxygen concentration of about 5.0 mg/L and a 12 h light/dark photoperiod. After acclimation for 2 weeks and pre-fasting for 24 h, the larvae were exposed to PS-NPs. The concentrations of PS-NPs were set as 5, 10, 25, and 50 mg/L among which the concentration of 50 mg/L was used in many related studies to reveal physiological mechanisms of toxicity of MPs or NPs (Brandts et al., 2018; Sendra et al., 2019; Yang et al., 2021). After 24 and 48 h of exposure, three to five fish larvae per concentration were randomly selected and whole-body sampled in liquid nitrogen for assays of metabolites and enzyme activities. According to the results of assays for energy metabolites and enzyme activities, three larvae in 50 mg/L were randomly selected and sampled after 48 h of exposure to PS-NPs to perform the transcriptome sequencing plus a control group. This process was repeated to prepare the samples for the validation of transcriptome sequencing.

### Assays for Energy Metabolites and Enzyme Activities

Samples were homogenized in phosphate-buffered solution and the supernatants were used for biochemical assays. The contents of glucose (Glu) and triglyceride (TG), and the activities of lactate dehydrogenase (LDH), pyruvate kinase (PK), aspartate aminotransferase (AST), alanine aminotransferase (ALT), superoxide dismutase (SOD), glutathione peroxidase (GPx) were determined using the diagnostic reagent kits (Nanjing Jiancheng, P.R. China) according to the product manuals. Briefly, the glucose content was measured using the glucose oxidase method and absorbance was measured at 505 nm (Kumar and Gill, 2018). The triglyceride content was measured by mixing tissue homogenates and enzyme reagents of 10× the volume and then measuring the absorbance at 510 nm. The activity of LDH was measured in the reaction wherein lactate is converted to pyruvate with NAD^+^ being the hydrogen donor. The activity of PK was determined by measuring the absorption value at 340 nm after using PK to catalyze the conversion of phosphoenolpyruvate to pyruvate and then to lactic acid. The activities of AST or ALT were determined according to the method described by Rietman and Frankel (1957). The SOD activity was measured using a cytochrome c reduction inhibition reaction in the xanthine-xanthine oxidase system and was determined by the absorption value at 550 nm (Bai et al., 2022). The GPx activity was measured by determining the change in the content of reduced glutathione in a redox reaction (Wang et al., 2019). The Coomassie blue method was used to determine the total protein level (Bradford, 1976).

### RNA Isolation and Sequencing

Total RNA was isolated from the whole-body samples using a miRNA isolation kit (Invitrogen, United States ). The concentration and integrity of total RNA were determined with a bioanalyzer (Nanodrop 2000; Thermo Scientific, United States) and agarose gel electrophoresis, respectively. The mRNA was isolated from total RNA using oligo(dT) magnetic beads and then broken into fragments. Double-stranded cDNAs were generated with reverse transcribed fragments followed by end repair and adapter attachment. After PCR amplification, the quality of the cDNA library was evaluated using an Agilent bioanalyzer (Agilent, United States) and then paired-end sequenced on the Illumina Hiseq X Ten platform.

Clean reads were obtained by removing reads containing poly-N and with low quality using Trimmomatic (v. 0.36) (Bolger et al., 2014). Clean reads were mapped to a reference genome using hisat2 (v. 2.2.1.0) (Kim et al., 2015). The websites to download the reference genome, mRNAs and information of genome annotation were shown in Supplementary Material S2.

Expression levels of genes were assessed as fragments per kilobase of exon million (FPKM) using Cufflinks (v. 2.2.1) (Trapnell et al., 2010; Roberts et al., 2011). For genes from the PS-NPs group and control group, FPKMs of each gene were used to conduct DEGs analysis with DESeq R package (v. 1.18.0). The thresholds for scanning DEGs were set as *P*-adjust < 0.05 and fold change >2. Gene Ontology (GO) enrichment and Kyoto Encyclopedia of Genes and Genomes (KEGG) pathway enrichment analysis were conducted based on DEGs using the R package. The thresholds for significant enrichment of GO or KEGG with DEGs were set as *P*-adjust < 0.05.

### Validation of Transcriptome Sequencing

To validate the DEGs from RNA-seq, changes in expression of several key DEGs, including proteasome 26S subunit, ATPase 3 (PSMC3), HSP70, ubiquitin specific peptidase 13 (USP13), DNA damage inducible transcript 4 (DDIT4), alcohol dehydrogenase 1 (ADH1), cytochrome P450 2D15 (P4502D15), fatty acid desaturase 2 (FADS2) and protein phosphatase 1 regulatory subunit 3B (PPP1R3B), were measured using real-time PCR. These genes are related to the response to protein or DNA damage, lipid metabolism, xenobiotics metabolism and energy metabolism, respectively. The primers for real-time PCR are shown in Supplementary Material S3. Real-time PCR was conducted on the Eppendorf Mastercycler Ep Realplex RT-PCR platform (Eppendorf, Germany) using TransStart Top Green qPCR SuperMix (Takara, Japan) according to the product manual. The 18s ribosomal RNA gene was used as the internal control gene and the relative gene expression level was calculated according to the 2^-∆∆Ct^ method (Schmittgen and Livak, 2008).

### Statistics

Concentrations of metabolites and enzyme activities were tested for normal distribution and homogeneity of variance first. If both were appropriate, the general linear model was employed to test the effects of exposure duration, PS-NPs concentration, and interaction on these parameters. Otherwise, the generalized linear model was used. Afterward, the one-way ANOVA or non-parametric test was used to test the effect of PS-NPs concentration on biochemistry indexes after different exposure periods. All the data of biochemistry assays were standardized based on the value of the control group and then were clustered. Gene expressions validated by real-time PCR were compared using a *t*-test. Data were represented as mean ± standard error. Statistical significance was set as *p* < 0.05. The statistical analysis was conducted with SPSS software (v. 17.0 IBM, United States) and the cluster was conducted with R software (v. 4.0.3).

## Results

### Changes in Energy Metabolites and Enzyme Activities

**FIGURE 1:**
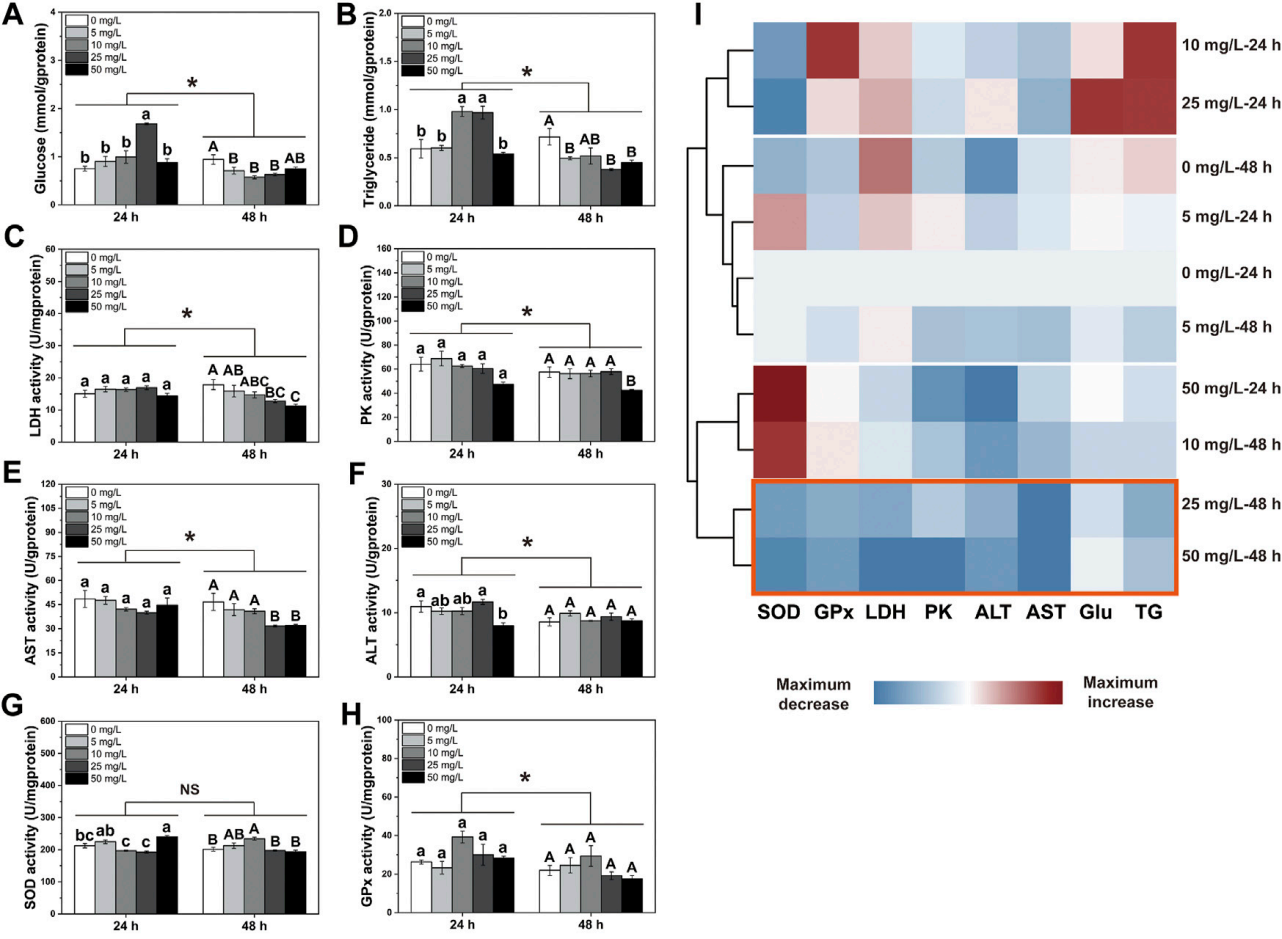
Changes in energy metabolism and antioxidant defense in the larval *Ietalurus punetaus* after exposure to polystyrene nanoplastics. **(A)** glucose content. **(B)** triglyceride content. **(C)** lactate dehydrogenase (LDH) activity. **(D)** pyruvate kinase (PK) activity. **(E)** aspartate aminotransferase (AST) activity. **(F)** alanine aminotransferase (ALT) activity. **(G)** superoxide dismutase (SOD) activity. **(H)** glutathione peroxidase (GPx) activity. **(I)** clustering analysis for indexes measured. Red box indicated the lowest levels of these metrics after exposure. Data are represented as mean ± standard error. Significant difference was set as *p* < 0.05.

Results showed that exposure durations and PS-NPs concentrations interacted on energy metabolites and enzyme activities except GPx, PK and AST activities (*P*_Inteaction-GPx_ = 0.32, *P*_Inteaction-PK_ = 0.503 and *P*_Inteaction-AST_ = 0.093, Figure 1). In general, after 48 h of exposure to 75-nm PS-NPs, all indexes except SOD activity showed lower levels than those after 24 h of exposure (*P*_tims-SOD_ = 0.122, Figure 1). After 24 h of exposure, 25 mg/L of PS-NPs increased the Glu content in the larval *I. punetaus* and, 10 mg/L and 25 mg/L PS-NPs increased the TG content (*P*_Glu_ < 0.001 and *P*_TG_ < 0.001, Figures 1A,B). The highest concentration of PS-MPs (50 mg/L) inhibited ALT activity but increased SOD activity after 24 h of exposure (*P*_ALT_ = 0.013 and *P*_SOD_ < 0.001, Figures 1F,G). After 48 h of exposure to 75-nm PS-NPs, the glucose content decreased in the groups exposed to 5, 10, and 25 mg/L of 75-nm PS-NPs and rebounded slightly in the highest-concentration group (*p* < 0.001, Figure 1A). Meanwhile, the TG content, LDH, PK and AST activities showed dose-dependent decreases with increased PS-NPs concentrations (*P*_TG_ = 0.003, *P*_LDH_ = 0.004, *P*_PK_ = 0.025 and *P*_AST_ = 0.004, Figures 1B–E). After 48 h of exposure, the SOD activity showed a peak in 10 mg/L PS-NPs and decreased to the control level with increased concentrations of PS-NPs (*p* < 0.001, Figure 1G). Activities of ALT and GPx was not affected by 48 h of exposure to PS-NPs (*P*_ALT_ = 0.223 and *P*_GPx_ = 0.125, Figures 1F,H). The clustering analysis showed that 48 h of exposure to higher concentrations (25 and 50 mg/L) of PS-NPs results in the lowest overall level for all indexes measured (*p* < 0.001, Figure 1I).

### Differentially Expressed Gene and Functional Analysis

**FIGURE 2:**
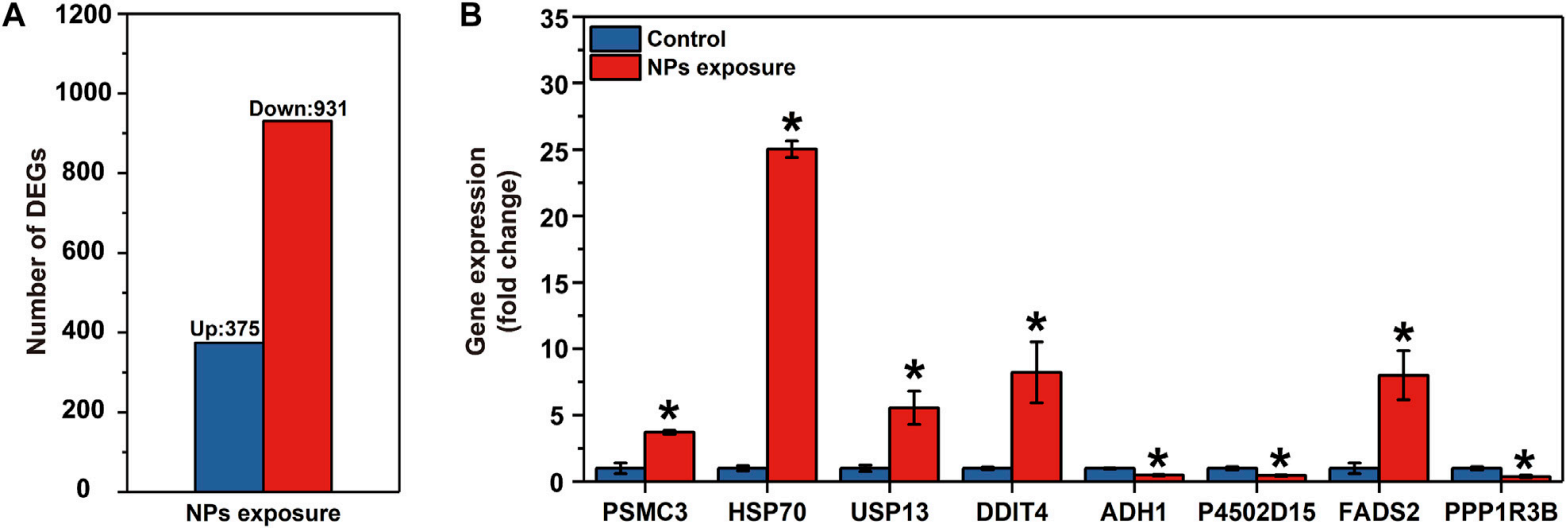
Results of the RNA-seq in the larval *Ietalurus punetaus* after 48 h of exposure to 50 mg/L polystyrene nanoplastics. **(A)** number of differentially expressed genes. **(B)** validation of key differentially expressed genes *via* real-time PCR. Data are represented as mean ± standard error. Star symbol indicates a significant change in gene expression between the exposure and control groups and significant threshold is set as *p* < 0.05.

Paired-end 150 bp sequencing resulted in 49.42 million reads per sample, and more than 48 million clean reads with a Q30 higher than 95% after quality control (Table 1). More than 95% of the clean reads were mapped to reference transcriptome and therein more than 90% were uniquely mapped. The transcriptomic analysis found 1,306 differentially expressed genes (DEGs), including 375 upregulated genes and 931 downregulated genes (Figure 2A and Supplementary Material S4).

**TABLE 1:** Results of sequencing read, quality control and mapping of each sample.

Sample Name	Raw reads (M)	Raw bases (G)	Clean reads (M)	Clean bases (G)	Q30 (%)	GC content (%)	Mapped reads (M)	Mapped ratio (%)	Uniquely mapped reads (M)	Uniquely mapped
										Ratio (%)
Control 1	49.42	7.41	48.09	7.08	95.23	48.88	46.20	96.08	43.67	90.82
Control 2	49.42	7.41	48.03	7.07	95.08	48.87	46.07	95.93	43.62	90.82
Control 3	49.42	7.41	48.10	7.09	95.17	48.94	46.30	96.27	43.61	90.69
NPs exposure 1	49.42	7.41	48.18	7.09	95.35	48.62	46.25	96.01	44.33	92.02
NPs exposure 2	49.42	7.41	48.16	7.08	95.31	48.34	46.08	95.69	33.18	91.75
NPs exposure 3	49.42	7.41	48.07	7.08	95.13	48.57	46.08	95.88	43.87	91.28

**FIGURE 3:**
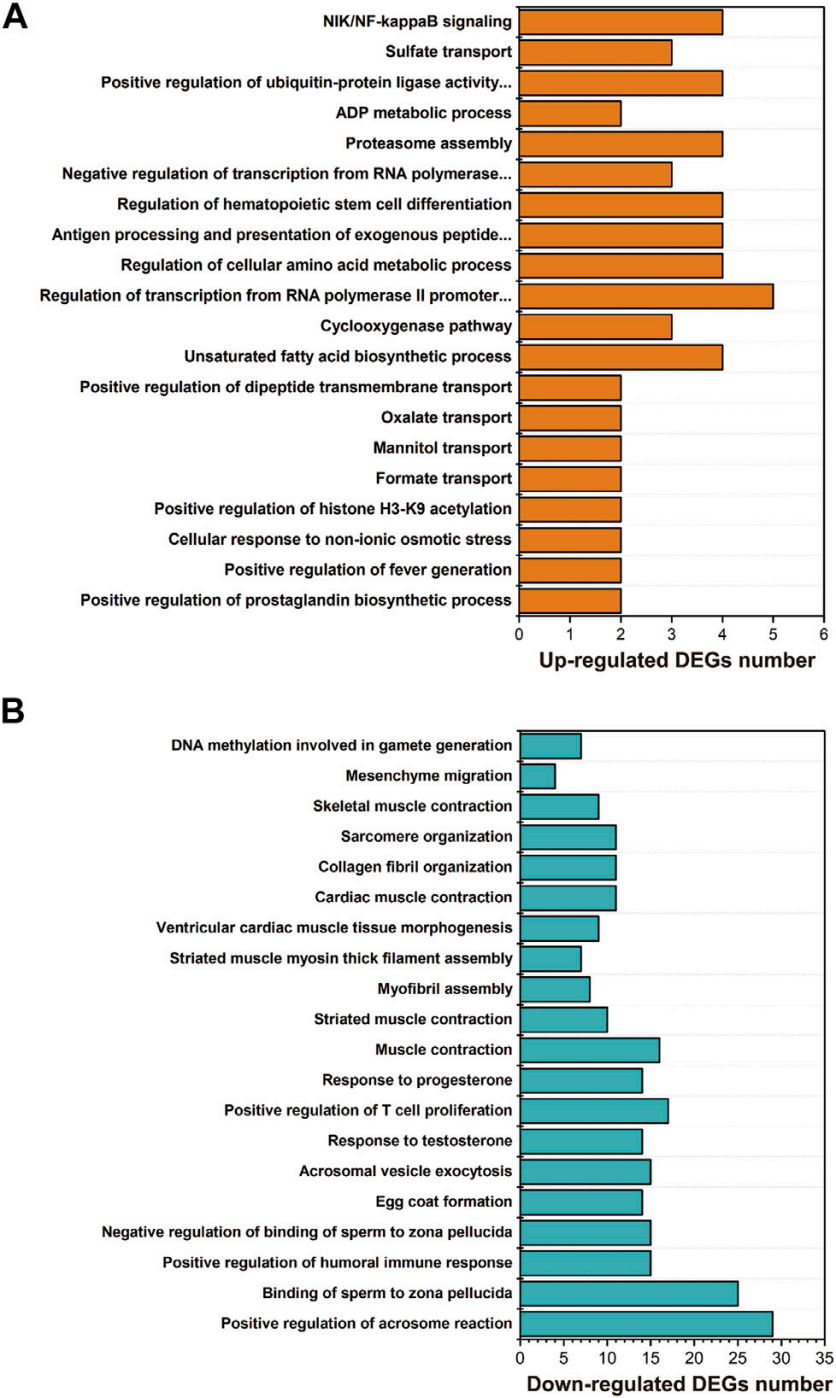
Results of GO enrichment with differentially expressed genes in the larval *Ietalurus punetaus* after 48 h of exposure to 50 mg/L polystyrene nanoplastics. **(A)** the biological process categories enriched with upregulated genes. **(B)** the biological process categories enriched with downregulated genes.

GO enrichment showed that the biological processes, including unsaturated fatty acid biosynthetic process (GO:0006636), lipid storage (GO:0019915), regulation of cellular amino acid metabolic process (GO:0006521), proteasome assembly (GO:0043248), response to unfolded protein (GO:0006986) or protein ubiquitination (GO:0016567), were significantly enriched with upregulated genes in larval *I. punetaus* after 48 h of exposure to 50 mg/L of PS-NPs (Figure 3A).

Several biological processes were significantly enriched with down-regulated genes in larval *I. punetaus* after 48 h of exposure to 50 mg/L of PS-NPs including those involved in gonad development [e.g. egg coat formation (GO:0035803), positive regulation of acrosome reaction (GO:2000344) or response to testosterone (GO:0033574)] and muscle contractility [muscle contraction (GO:0006936), striated muscle contraction (GO:0006941) and skeletal muscle contraction (GO:0003009)] (Figure 3B).

**FIGURE 4:**
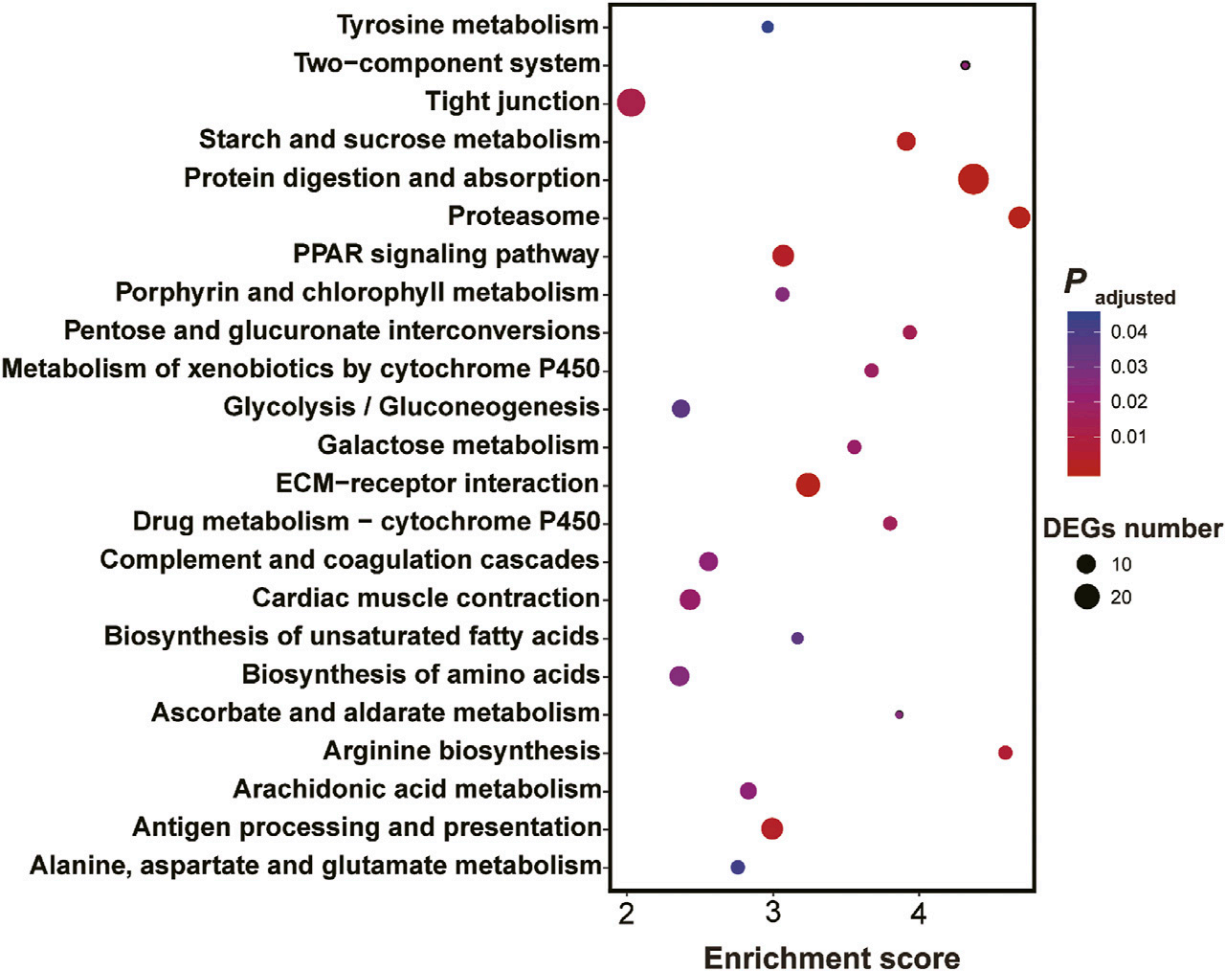
Results of KEGG enrichment with differentially expressed genes in the larval *Ietalurus punetaus* after 48 h of exposure to 50 mg/L polystyrene nanoplastics.

KEGG enrichment showed the pathways which were significantly enriched with DEGs were related to the biological functions revealed by GO enrichment. These pathways included proteasome (ko03050), peroxisome proliferator-activated receptors (PPAR) signaling pathway (ko03320), biosynthesis of unsaturated fatty acids (ko01040), protein digestion and absorption (ko04974), drug metabolism-cytochrome P450 (ko00982) and metabolism of xenobiotics by cytochrome P450 (ko00980) and serval pathways involved in amino acid metabolism (Figure 4).

### Key DEGs in the Important Pathways

**FIGURE 5:**
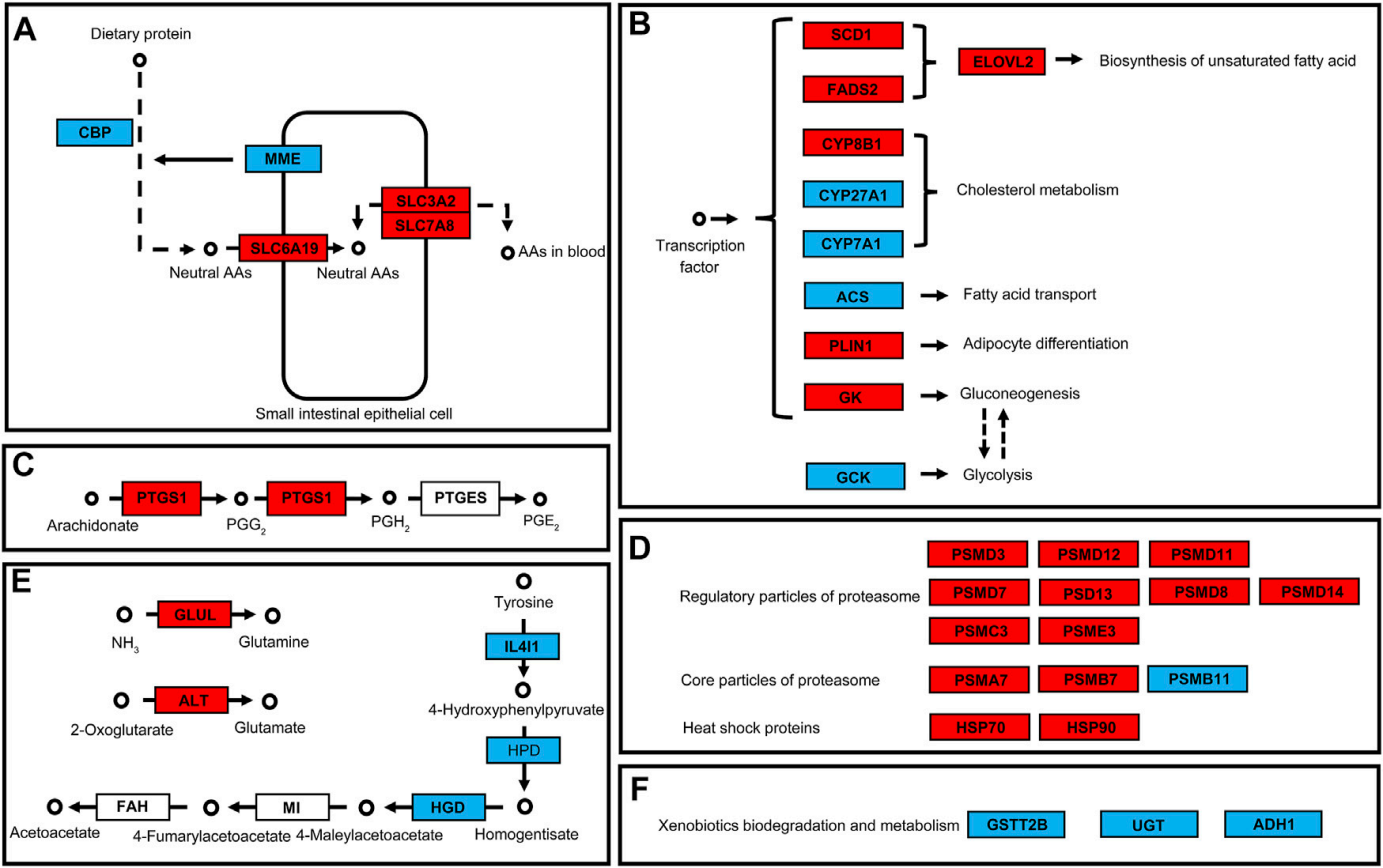
Effects of polystyrene nanoplastics exposure on the key differentially expressed genes in the pathways of the *Ietalurus punetaus* larvae. **(A)** protein digestion and absorption. **(B)** PPAR pathway and downstream genes. **(C)** biosynthesis of prostaglandins. **(D)** changes in genes related to proteasomes and heat shock proteins. **(E)** biosynthesis of specific amino acids. **(F)** changes in expression of several genes involved in xenobiotics metabolism. Up-regulated genes are marked in red and down-regulated genes are marked in blue.

In the protein digestion and absorption pathway, two genes involved in protein digestion were downregulated while three amino acid transport channel genes were upregulated after 48 h of exposure to 50 mg/L of PS-NPs (Figure 5A). In the PPAR signaling pathway, expressions of multiple downstream genes were affected by 48 h of exposure to 75-nm PS-NPs. Stearoyl-CoA desaturase 1 (SCD1) and fatty acid desaturase 2 (FADS2), which participate in lipid metabolism, were upregulated and the downstream gene, elongation of very long chain fatty acids protein 2 (ELOVL2), were activated to enhance the biosynthesis of unsaturated fatty acid (Figure 5B). Glycerol kinase (GK) which participates in gluconeogenesis was activated after 48 h of exposure to 50 mg/L of PS-NPs but the expression of glucokinase (GCK) which regulates glycolysis was depressed (Figure 5B). In the arachidonic acid metabolism pathway, prostaglandin-endoperoxide synthase 1 (PTGS1) which regulates the biosynthesis of prostaglandin H_2_ and G_2_ was activated in larval *I. punetaus* by 48 h of exposure to 50 mg/L of PS-NPs (Figure 5C). In the proteasome pathway, multiple genes of subunits forming regulatory particles or core particles of proteasome were up-regulated in larval *I. punetaus* after 48 h of exposure to 50 mg/L of PS-NPs (Figure 5D). At the same time, expressions of two heat shock proteins (HSPs) increased (Figure 5D). The pathway to synthesize acetoacetate from tyrosine had three down-regulated genes including interleukin 4 induced 1 (IL4I1), 4-hydroxyphenylpyruvate dioxygenase (HPD) and homogentisate 1,2-dioxygenase (HGD) (Figure 5E). Glutamate-ammonia ligase (GLUL) and alanine transaminase (ALT) which take part in the biosynthesis of glutamine or glutamate were activated in the larval *I. punetaus* after 48 h of exposure to 50 mg/L of PS-NPs (Figure 5E). The genes involved in xenobiotics metabolism, including glutathione S-transferase theta 2B (GSTT2B), glucuronosyltransferase (UGT), and alcohol dehydrogenase 1 (ADH1), were depressed by 48 h of exposure to PS-NPs (Figure 5F).

### Validation by Real-Time PCR

The mRNA levels of PSMC3, HSP70, USP13, DDIT4 and FADS2 were significantly increased in *I. punetaus* larvae after 48 h exposure to 50 mg/L of PS-NPs (*P*_PSMC3_ = 0.003, *P*_HSP70_ < 0.001, *P*_USP13_ = 0.023, *P*_DDIT4_ = 0.034 and *P*_FADS2_ = 0.02, Figure 2B)*.* The other three genes were down-regulated in *I. punetaus* larvae after 48 h exposure to PS-NPs (*P*_ADH1_ = 0.005, *P*_P4502D15_ = 0.018 and *P*_PPP1R3B_ = 0.021, Figure 2B).

## Discussion

Fish play an important role in aquatic ecosystems and are valuable fishery resources. Revealing the toxicological consequences of plastic pollution on fish especially their early life-history stage is of great significance. Previous studies showed that microplastics and nanoplastics can impair the locomotion, energy reserves or other physiological functions of fish adults (Mattsson et al., 2015; Mattsson et al., 2017; Yin et al., 2018; Yin et al., 2019). In the present study, our results indicated that the 75-nm PS-NPs impacted energy metabolism and several important molecular pathways, including protein digestion, response to damaged protein, gonad development and muscle contraction, in the larval channel catfish *Ietalurus punetaus*.

Exposure to PS-NPs affected energy metabolism in the larval *I. punetaus* and the influences were dose- and exposure duration-dependent. After 24 h of exposure, increased contents of glucose and triglyceride, which are both important sources of energy, indicated that exposure to 75-nm PS-NPs may disturb the utilization of these metabolites and lead to accumulation. These were similar to the findings that contents of glucose or triglyceride increased in juveniles or adults of several fish species after exposure to microplastics (Lu et al., 2016; Hamed et al., 2019). After 48 h of exposure, decreased contents of these two metabolites and the activated PPAR pathway suggested an urgent energy demand and rapid consumption of energy in larval *I. punetaus* encountering 75-nm PS-NPs. Decreased glucose was also reported in the larval zebrafish after 48 h of exposure to 25-nm PS-NPs, indicating the sensibility of fish larvae to NPs (Brun et al., 2019). Meanwhile, 48 h of exposure to high concentrations of PS-NPs inhibited the anaerobic metabolism in the larval *I. punetaus*, suggested by decreased LDH and PK activities. Anaerobic metabolism is important for aquatic organisms to offer sufficient energy during exposure to plastic pollution (Barboza et al., 2018; Li Y. et al., 2020). The inhibition of PS-NPs on anaerobic metabolism of the larval *I. punetaus* may amplify the adverse effects of the energy shortage and in turn affect other physiological functions. The relatively lower activities of ALT and AST, which play the important role in amino acid, glucose, or long-chain free fatty acid metabolisms, also indicated the inhibition of PS-NPs on the metabolism of the larval *I. punetaus* (Sookoian and Pirola, 2015). The activities of ALT and AST are expected to increase in the aquatic organisms exposed to small plastic particles as the indicators of plastic toxicity (Banaee et al., 2019; Hamed et al., 2019). However, our results suggested that inhibition on ALT and AST activities may also be a part of NPs toxicity to the larval fish and differ from the effects on adults.

Increased reactive oxygen species (ROS) were recognized as one of the toxic effects of MPs or NPs on organisms (Jeong et al., 2016; Liu et al., 2020). Excess ROS can damage biomacromolecules especially when the antioxidant defense is overwhelmed (Costantini et al., 2010). In the present study, our results showed that PS-NPs caused few effects on SOD and GPx activities in the larval *I. punetaus*. After 24 h of exposure, the SOD activity in the larval *I*. *punetaus* increased in the highest concentration of PS-NPs but increased in the 10 mg/L after 48 h of exposure, indicating the changes in sensitivity of SOD activity with different exposure duration and a potential accumulative effect of PS-NPs. Dramatic changes in antioxidant enzyme activities, including SOD and GPx, were observed in fish species after long-term exposure to MPs or NPs, such as 7 or 21 days of exposure (Qiao et al., 2019; Li et al., 2021). This may mean that the effects of NPs on antioxidant defense depend on relatively long exposure duration.

The transcriptomic analysis showed that 48 h of exposure to 50 mg/L of 75-nm PS-NPs disturbed the digestive process and induced the responses to protein damage. Ingested MPs or NPs can lead to blockage of the digestive tract and impaired intestinal health in organisms (Cole et al., 2013; Gu et al., 2020). In the larval *I. punetaus*, inhibited expression of carboxypeptidase B (CPB) and membrane metalloendopeptidase (MME), which convert dietary protein to peptides and amino acids, suggested impaired protein digestion after 48 h of exposure (Vetel et al., 2000; Bayés et al., 2006). Expressions of three amino acid transporters increased to enhance or maintain the transport of neutral amino acids as the potential response to digestive disorders (Böhmer et al., 2010; Ikeda et al., 2017; Wu et al., 2017). The effects on the digestive process may decrease energy intake and lead to the low level of energy reserves. The upregulation of proteasome-related genes and heat shock proteins (HSPs) may be due to the damage caused by PS-NPs to proteins and their upregulation can help maintain protein structure or clear damaged proteins (Morimoto and Santoro, 1998; Shang and Taylor, 2011). Nanoplastics can interact with proteins to form corona and damage the protein structures when they enter the biological fluids of organisms (Mattsson et al., 2015; Canesi et al., 2017). The repair or removal of damaged proteins *via* proteasomes or HSPs is a process that consumes huge energy (Zhou et al., 2004; Viana et al., 2008). This process in the larval *I. punetaus* exposed to PS-NPs may result in a further reduction in the contents of energy metabolites.

As one of the responses to the effects of PS-NPs, the larval *I. punetaus* activated genes that regulate the synthesis of prostaglandins or specific amino acids. The PTGS1, also known as COX1, participates in the biosynthesis of prostaglandins or cellular housekeeping response and can provide prostaglandins in the digestive tract to maintain the integrity of the mucosal epithelium or maintain cell survival and proliferation (Otto and Smith, 1995; Cohn et al., 1997). This may be a protective mechanism for the digestive tract of the larval *I. punetaus* against the PS-NPs. Similarly, activated GLUL suggested a need for glutamine in the larval *I. punetaus* after exposure to PS-NPs. Glutamine is an important energy substrate and can regulate the expression of genes involved in nutrient metabolism, protein hemostasis and cell survival (Xi et al., 2011). Studies have reported that chronic exposure to 44-nm PS-NPs consumed glutamine in shrimp *Litopenaeus vannamei*, proving the need for glutamine in aquatic organisms exposed to NPs (Chae et al., 2019).

Exposure to PS-NPs also affected the expression of genes involved in gonad development and muscle contraction in the larval *I. punetaus*. This may be due to the direct toxicity of PS-NPs or partly due to impaired energy metabolism. Gonad development depends on sufficient energy intake and supply, and exposure to high concentrations of PS-NPs may thus affect the gonad development of the larval *I. punetaus* (Pérez Camacho et al., 2003). Exposure to PS-NPs can affect multiple locomotion behaviors in many fish species. For example, NPs-exposed crucian carp *Carassius* showed reduced explorative behaviors (Mattsson et al., 2015). Usually, the adverse effects on behaviors are related to alteration in energy metabolism capacity and a direct effect of NPs on the fish brain (Mattsson et al., 2015). In the larval *I. punetaus* exposed to 75-nm PS-NPs for 48 h, our results suggested that PS-NPs exposure can alter the expression of genes involved in muscle contraction, which may potentially link with impaired energy metabolism.

Overall, the present study revealed that acute exposure to 75-nm PS-NPs disrupted the energy metabolism and impacted a series of molecular pathways in the larval *I. punetaus*. Exposure to 75-nm PS-NPs for 48 h decreased the contents of energy metabolites and inhibited the activities of various metabolic enzymes. Meanwhile, 75-nm PS-NPs can alter the protein digestive process and induce responses to protein damage in the larval *I. punetaus*. Genes involved in gonad development and muscle contraction were depressed in the larval *I. punetaus* after 48 h of exposure to 75-nm PS-NPs. As these effects occur during the critical phase of the development of fish, exposing the fish larvae to NPs may therefore cause non-negligible effects on the reproduction, behaviors and survival of fish adults and potential influences on individual fitness or population structure. Our study provided a perspective on the toxicological consequences of PS-NPs to the larval fish especially energy metabolism and molecular pathways.

## Data Availability

The datasets presented in this study can be found in online repository. The name of the repository and accession number(s) can be found as bellow: Dryad Digital Repository https://doi.org/10.5061/dryad.np5hqbzwb.
