# Supplementary material for: Effects of Acute Exposure to Polystyrene Nanoplastics on the Channel Catfish Larvae: Insights From Energy Metabolism and Transcriptomic Analysis
Source: Front Physiol. 2022 Jun 1;13:923278. doi: 10.3389/fphys.2022.923278 (PMC9198484; doi:10.3389/fphys.2022.923278)
Supplement: Supplementary file 3 [file Table1.DOCX]

**Effects of acute exposure to polystyrene nanoplastics on the channel catfish larvae: Insights from energy metabolism and transcriptomic analysis**

Websites to download the information about the reference genome

Genome Database: ftp://ftp.ncbi.nlm.nih.gov/genomes/all/GCF/001/660/625/GCF_001660625.1_IpCoco_1.2/GCF_001660625.1_IpCoco_1.2_genomic.fna.gz

mRNA Database: ftp://ftp.ncbi.nlm.nih.gov/genomes/all/GCF/001/660/625/GCF_001660625.1_IpCoco_1.2/GCF_001660625.1_IpCoco_1.2_rna.fna.gz

Genome annotation file: ftp://ftp.ncbi.nlm.nih.gov/genomes/all/GCF/001/660/625/GCF_001660625.1_IpCoco_1.2/GCF_001660625.1_IpCoco_1.2_genomic.gff.gz
