# Supplementary figures and images for: Effects of Acute Exposure to Polystyrene Nanoplastics on the Channel Catfish Larvae: Insights From Energy Metabolism and Transcriptomic Analysis
Source: Front Physiol. 2022 Jun 1;13:923278. doi: 10.3389/fphys.2022.923278 (PMC9198484; doi:10.3389/fphys.2022.923278)

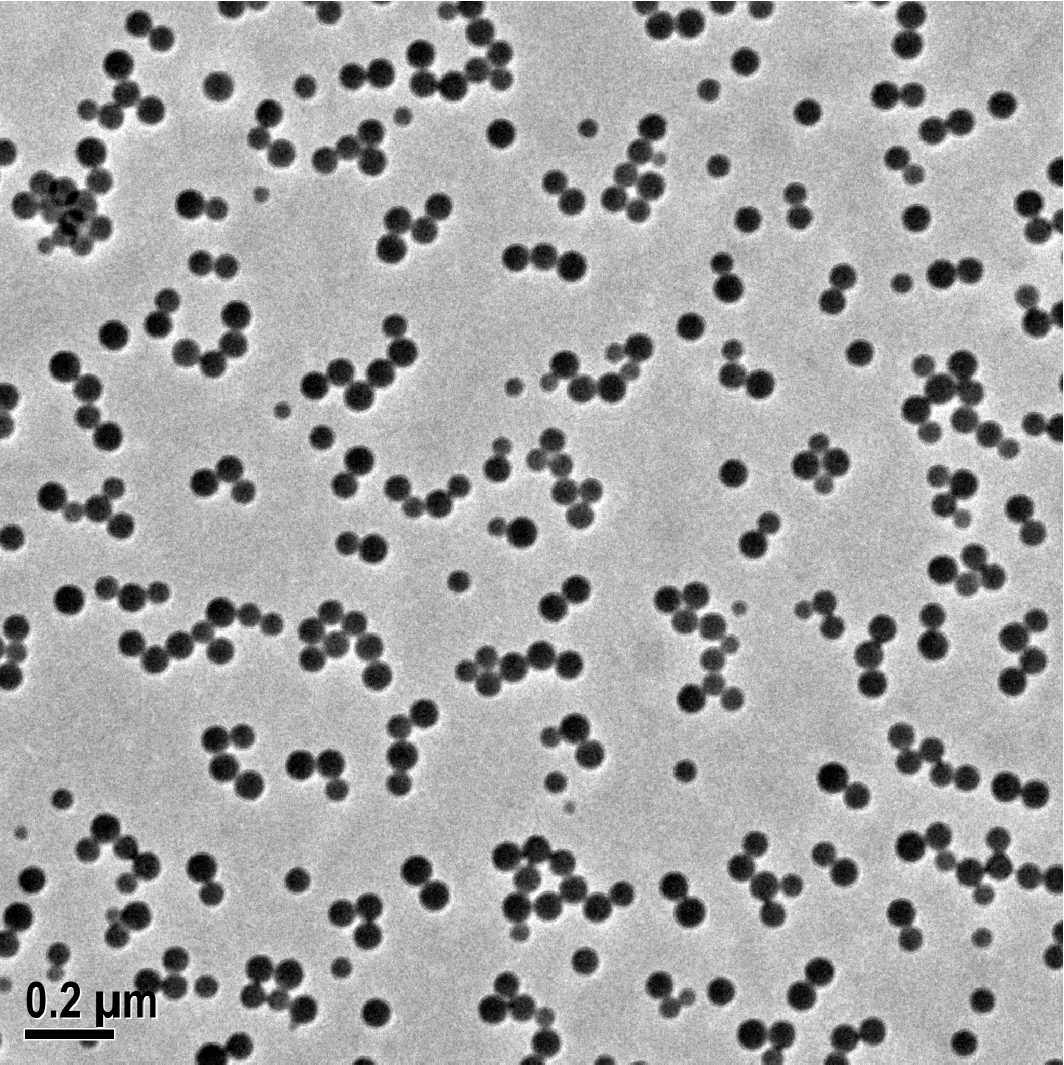

Supplement: Supplementary file 4 [file Image1.TIF]
